# High-quality bonds: *serine acetyltransferase 2* gene revealed by GWAS is associated with grain protein content in spring durum wheat

**DOI:** 10.3389/fpls.2025.1632673

**Published:** 2025-08-12

**Authors:** Aleksey Ermolaev, Ludmila Bespalova, Varvara Korobkova, Aleksey Yanovsky, Lubov Nazarova, Aleksandra Kroupina, Anastasia Chernook, Aleksandra Mudrova, Viktoria Voronezhskaya, Pavel Kroupin, Gennady Karlov, Mikhail Divashuk

**Affiliations:** ^1^ All-Russian Research Institute of Agricultural Biotechnology, Moscow, Russia; ^2^ P.P. Lukyanenko National Grain Center, Krasnodar, Russia; ^3^ Moscow Institute of Physics and Technology, Moscow, Russia; ^4^ National Research Center “Kurchatov Institute”, Moscow, Russia

**Keywords:** grain protein content, spring durum wheat, GWAS, *serine acetyltransferase 2*, KASP, SNP, molecular marker

## Abstract

Grain protein content (GPC) is a critical determinant of durum wheat quality, with cysteine playing a pivotal role in gluten strength. This study aimed to develop genetic markers associated with GPC through a genome-wide association study (GWAS) and validate their utility for breeding programs. A panel of 190 durum wheat accessions was phenotyped for GPC across multiple environments and genotyped using 4927 high-quality SNPs. GWAS identified a significant SNP on chromosome 4B, located in an intergenic region. Through the analysis of linkage disequilibrium decay rate, and functional gene ontology annotation, the *serine acetyltransferase 2* gene involved in cysteine biosynthesis was identified as a candidate gene for GPC. A missense mutation (Gly325Ser) in the ninth exon of *sat2* was associated with a 1.33% GPC increase in spring durum wheat recombinant inbred lines. Structural analysis indicated that the Gly325Ser mutation alters the SAT2 protein’s C-terminal *α*-helix, potentially influencing enzyme activity. Additionally, an intronic SNP showed association with multi-year average GPC increase of 0.92% in spring durum wheat. Despite the intronic SNP’s lack of direct amino acid impact, its high phenotypic variance explained (40.23% in spring wheat) suggests regulatory roles in gene expression. Expression profiling of *TAsat2* homologous from bread wheat revealed peak transcription during grain filling stages, aligning with grain protein accumulation dynamics. The developed KASP markers demonstrated robust allelic discrimination, offering practical tools for marker-assisted selection. This study provides actionable genetic resources for breeding high-protein spring durum wheat genotypes.

## 1 Introduction

Durum wheat (*Triticum turgidum* L. subsp. durum (Desf.) Husn.) is one of the most important grain crops in the global food industry. The quality of durum wheat grain largely depends on grain protein content (GPC), which is a key determinant of pasta and cereal quality (Sissons et al., 2021; Fu et al., 2018; Kaplan Evlice, 2022). A crucial component of durum wheat grain protein is the amino acid cysteine, which enhances gluten strength by forming disulfide bonds that contribute to the three-dimensional structure of dough. Although cysteine constitutes only 2% of gluten amino acids, it is essential for optimal dough processing characteristics (Gasparre and Rosell, 2023; Gao et al., 2013). Therefore, identifying genes involved in cysteine metabolism and developing molecular markers linked to increased GPC could facilitate the breeding of elite wheat varieties with superior grain quality.

GPC is a complex trait regulated by multiple quantitative trait loci (QTLs) that interact with each other and with environmental factors (Nigro et al., 2019). However, not all major QTLs are stable across diverse environments, limiting their utility in marker-assisted selection (Kumar et al., 2018). QTLs and candidate genes influencing GPC have been identified on nearly all chromosomes in both cultivated and wild wheat (Giancaspro et al., 2019). The most significant QTL, associated with the majority of phenotypic variation in GPC, was mapped to the short arm of chromosome 6B in wild tetraploid wheat (*T. turgidum* spp. dicoccoides) (Uauy et al., 2006). Subsequent studies identified 325 main-effect QTLs and 42 epistatic QTLs (Kumar et al., 2018). Within the major 6B QTL, the functional gene *Gpc-B1* was cloned; it encodes the NAC transcription factor (NAM-B1), which accelerates plant senescence and enhances nitrogen remobilization from leaves to developing grains (Uauy et al., 2006). Other genes, including *Asparagine synthetase 1*, *Glutamine synthetase 1*, *NPF*, and *NRT*, have also been implicated in nitrogen uptake and remobilization, further influencing GPC (Balyan et al., 2016). Thus, most known GPC-associated genes in wheat are involved in nitrogen metabolism.

Sulfur is another critical component of proteins, as it is incorporated into the amino acids methionine and cysteine. In durum wheat storage proteins, sulfur is essential for synthesizing sulfur-containing amino acids like cysteine, which form disulfide bonds between cysteine residues, determining the physical properties of dough (Islam et al., 2019; Shewry, 2011). Consequently, identifying genes related to sulfur metabolism and developing molecular markers for increased sulfur content could improve the quality of durum wheat-derived products by enhancing flour properties.

The aim of this work was to identify genomic loci associated with GPC in durum wheat grain and to develop molecular markers for breeding durum wheat for high GPC. We employed a genome-wide association study (GWAS) to identify a novel target gene, *sat2*, for improving sulfur utilization and GPC in durum wheat breeding programs. As a result, Kompetitive Allele-Specific PCR (KASP) markers were developed for the polymorphism associated with higher GPC.

## 2 Materials and methods

### 2.1 Plant materials and field experiments

A total of 190 accessions of spring and winter durum wheat were used for the study. These include commercial cultivars and promising candidate lines for registration from the P.P. Lukyanenko National Grain Center (Krasnodar, Russia). Field experiments with a panel of winter and spring accessions of durum wheat were carried out across three growing seasons (2019–2020, 2020–2021, and 2021–2022) at two different locations — 45^°^01’45.7”N and 38^°^52’48.0”E (Krasnodar, Russia), and 45^°^08’52.0”N and 37^°^43’02.9”E (Kuban, Russia), using methods previously described (Kroupin et al., 2023; Kroupina et al., 2023).

For the evaluation of developed KASP markers, an expanded data set was used. In addition to the 190 accessions of spring and winter durum wheat used for GWAS, an additional 40 spring and winter durum wheat accessions were used in order to improve the statistical power of the analysis. Field experiments for these additional 40 accessions were conducted in the same manner as for 190 accessions, using methods previously described (Kroupin et al., 2023; Kroupina et al., 2023).

A panel of 43 recombinant inbred lines (RILs) of spring durum wheat used for KASP markers evaluation was created via a previously described protocol (Chernook et al., 2019). The field experiments were conducted in Moscow (55^°^50’N, 37^°^33’E) and Krasnodar (45^°^41’N, 38^°^55’E) in 2018 and 2021. The experiments were carried out under field conditions following the adopted methodology (Chernook et al., 2019).

### 2.2 Phenotyping

GPC in durum wheat panel was measured in 500 grams of grain using the Infratec™1241 grain analyzer (Foss Analytical, Hillerød, Denmark) following the manufacturer’s protocol. GPC was measured separately for each wheat accession in each environmental condition (growing season and location). Broad-sense heritability (*H* ^2^) was calculated as the proportion of genotype variance to total variance using the following formula:


(1) $$H^{2}=\frac{\sigma_{g}^{2}}{\sigma_{g}^{2}+\sigma_{ge}^{2}n_{e}^{-1}+\sigma_{\epsilon }^{2}n_{e}^{-1}}$$

Where $\sigma_{g}^{2}$ denotes the genetic variance, $\sigma_{ge}^{2}$ denotes the variance of the interaction between the genotype and the environment, $\sigma_{\epsilon }^{2}$ denotes the residual variance *n_e_* denotes the number of environments.

GPC in durum wheat RILs was measured in 50 grams of grain using the InfraLUM FT-12 grain analyzer (Lumex, Saint Petersburg, Russia) following the manufacturer’s protocol. GPC was measured separately for each RIL accession in each environmental condition (growing season and location).

### 2.3 Genotyping and SNP calling

Genotyping for all 190 durum wheat accessions was carried out using the Breeders’ 35K Axiom^®^ array. Total genomic DNA for genotyping was isolated from four-day-old seedlings using the CTAB method (Murray and Thompson, 1980). Signals from the SNP microarray were converted into genotype calls using Axiom Analysis Suite Software 4.0 (Thermo Fisher Scientific, Inc.), with standard settings for a diploid organism. Only A- and B-subgenome variants were called. OTVs (off-target variants) were processed using the OTV-caller utility as part of the Axiom Analysis Suite Software 4.0 program. SNP metrics after OTV-caller were regenerated. Only variants of the PolyHighResolution category (polymorphic SNPs that showed good separation of the signal from different alleles) belonging to the A- and B-sub genomes were used for analysis. Further filtering was performed using plink2 v2.00a5LM (Chang et al., 2015). SNPs containing more than 10% of missed genotypes, as well as SNPs with MAF (Minor Allele Frequency) less than 5%, were removed as variants in which polymorphism may be a result of genotyping error. The final set of SNPs consisted of 4927 variants, which were used for GWAS.

### 2.4 Genome-wide association analysis

The Bayesian-information and Linkage-disequilibrium Iteratively Nested Keyway (BLINK) algorithm implemented in the R package GAPIT version 3.1.0 was used to conduct GWAS (Huang et al., 2019). The first 10 principal components of population structure were calculated using plink2 v2.00a5LM (Chang et al., 2015) and were included in GWAS as covariates to account for population structure as a fixed effect. The first two principal components accounting for the majority of the explained variance were used for visualization of population structure. The kinship matrix (K) was calculated using the VanRaden method (VanRaden, 2008) and used to account for genetic relatedness as a random effect. BLUPs (Best Linear Unbiased Prediction) for each cultivar in each year were calculated for GPC using the R package “lme4” (Bates et al., 2015) using the following formula:


(2) $$Y_{ijkl}=\mu +\nu_{i}+y_{j}+t_{k}+s_{l}+e_{ijkl}$$

where *Y_ijkl_* denotes the average GPC for variety *i* of type *k* collected in *l* in year *j*, *µ* denotes the overall average, *ν_i_* denotes random effect of genotype *i*, *y_j_* denotes random effect of year *j*, *t_k_* denotes random effect of durum wheat growth habits *k*, *s_l_* is random effect of location *l*, and *e_ijkl_* is the residual error associated with variety *i* of type *k* collected in *l* in year *j*. The vector of coefficients corresponding to *ν_i_* (BLUPs) was utilized as the phenotype for GWAS. The resulting P-values were adjusted using the VIF (variance inflation factor) (Tsepilov et al., 2013). To visually assess the deviation between the observed and expected P-value distributions, a Quantile-Quantile (Q-Q) plot was used. The genome-wide inflation factor *λ* was calculated to analytically assess the deviation between the observed and expected P-value distributions (Dadd et al., 2009). To identify statistically significant SNPs, an FDR (False Discovery Rate) threshold of 5% was applied. Statistical analysis and result visualization were conducted using R version 4.3.1.

### 2.5 Identification of the candidate genes

The coordinates of statistically significant SNPs in the durum wheat reference genome assembly Svevo.v1 were found based on previously mapped probe oligos from the Breeders’ 35K Axiom^®^ array to the durum wheat genome assembly (Maccaferri et al., 2019). The search area for candidate genes was limited on either side of the statistically significant SNP by the average distance at which linkage disequilibrium (LD) becomes indistinguishable from the background LD. This approach was chosen due to the fact that the Breeders’ 35K Axiom^®^ array was originally developed for bread wheat, which does not guarantee preservation of the order of markers being used for durum wheat genotyping, and also because of the large number of chromosomal rearrangements shown in durum wheat, which can disrupt unpredictably the real order of markers in different varieties (Badaeva et al., 2007). Thus, a genome-wide threshold for reducing LD to background levels appears to be an adequate alternative to searching within local LD block. The selection of candidate genes was based on the existing gene ontology (GO) annotation in the durum wheat genome assembly Svevo.v1 (Maccaferri et al., 2015).

### 2.6 Mining of polymorphisms in candidate gene and design of KASP markers

To investigate the genetic diversity, the candidate gene was sequenced in six durum wheat accessions utilized for GWAS. For this purpose, primers were developed for the ORF (open reading frame) of the gene, resulting in overlapping PCR products ranging from 500 to 700 bp. Primers were designed using Primer3Plus (Untergasser et al., 2012). Sequencing was performed on pairedend 150 bp reads. The libraries were prepared using the SG GM Maxi Plus kit (Raissol, Moscow). Sequencing was performed using a FASTASeq 300 sequencer (Genomed, Moscow). The quality of the obtained reads was evaluated with FastQC v0.12.1 (www.bioinformatics.babraham.ac.uk/projects/fastqc/, accessed on January 10, 2024). The reads were trimmed using bbduk v39.06, which is part of the BBTools toolkit (www.sourceforge.net/projects/bbmap, accessed on January 10, 2024). Reads were mapped to the reference sequence of the gene from the durum wheat genome assembly Svevo.v1 (Maccaferri et al., 2019) using bowtie2 v2.5.2 (Langmead and Salzberg, 2012) with default parameters, except –dovetail. PCR and optical duplicates were removed using samtools v1.18 (Danecek et al., 2021). Mutations were identified using freebayes v1.3.6 (Garrison and Marth, 2012). When identifying variants, the top 4 alleles were accounted for based on the sum of nucleotide read quality (-n 4). The low-quality SNPs (phred score <20) were removed using vcftools v0.1.16 (Danecek et al., 2021). The consensus gene sequence incorporating the identified mutations was obtained using bcftools v1.18 (Danecek et al., 2021). Multiple sequence alignment was conducted using Clustal Omega v1.2.4 (https://www.ebi.ac.uk/Tools/msa/clustalo/, accessed on January 10, 2024) and visualized using Jalview 2.11.4.1 (Waterhouse et al., 2009).

For identified SNPs, discriminatory primers were developed using the Primer3Plus program (Untergasser et al., 2012), the 3’ nucleotide of which is complementary to one of the alleles. To develop markers, either exonic non-synonymous SNPs were used or intronic SNPs.

### 2.7 Association between the developed KASP markers and GPC

The analysis was conducted on a complete dataset and separately on collections of winter and spring durum wheat. Data on GPC for 2023 were also included in the analysis. To assess the presence of an association, we employed a mixed linear model (MLM) of the “lme4” package (Bates et al., 2015) with random slopes and intercepts. For a complete dataset, both a growth habit (winter or spring) and a year were accounted for as random effects. In the separate dataset for spring and winter durum wheat, the year was treated as a random effect. The KASP marker allelic state served as a fixed effect in both complete and separate datasets. We assessed the statistical significance of the association between allelic state and phenotype using type 2 sum of squares analysis of variance (ANOVA), with a significance threshold of *α* = 0.05. PVE (Percent of Variance Explained) for RILs was calculated using the following formula:


(3) $$PVE=2f(1-f)\beta^{2}$$

where *f* denotes the MAF and *β* denotes effect size (the slope of the linear model). PVE values were expressed as percentages. PVE for extended GWAS panel was calculated accounting for population structure using MLM. The first 10 principal components of population structure and GRM (Genomic Relationship Matrix) were calculated using plink2 v2.00a5LM (Chang et al., 2015) and were included as covariates (fixed effect) and genetic relationship estimator (random effect). PVE was estimated using REML (REstricted Maximum Likelihood) from “lme4” R package (Bates et al., 2015) as fraction genetic variance in total variance.

To assess the presence of an association between the genotype of the KASP marker and GPC, MLM from the “lme4” package (Bates et al., 2015) was used with a random slope and intercept. Technical replicates were considered random effects. To calculate the statistical significance of the association between allelic state and GPC, an ANOVA (type 2 sum of squares) was performed at the significance level of *α* = 0.05. PVE was calculated using formula 3. Statistical analysis and result visualization were conducted using R version 4.3.1.

## 3 Results

### 3.1 GWAS using polymorphic population enables to find candidate gene *sat2* and its allelic variant with altered protein structure

**Figure 1:**
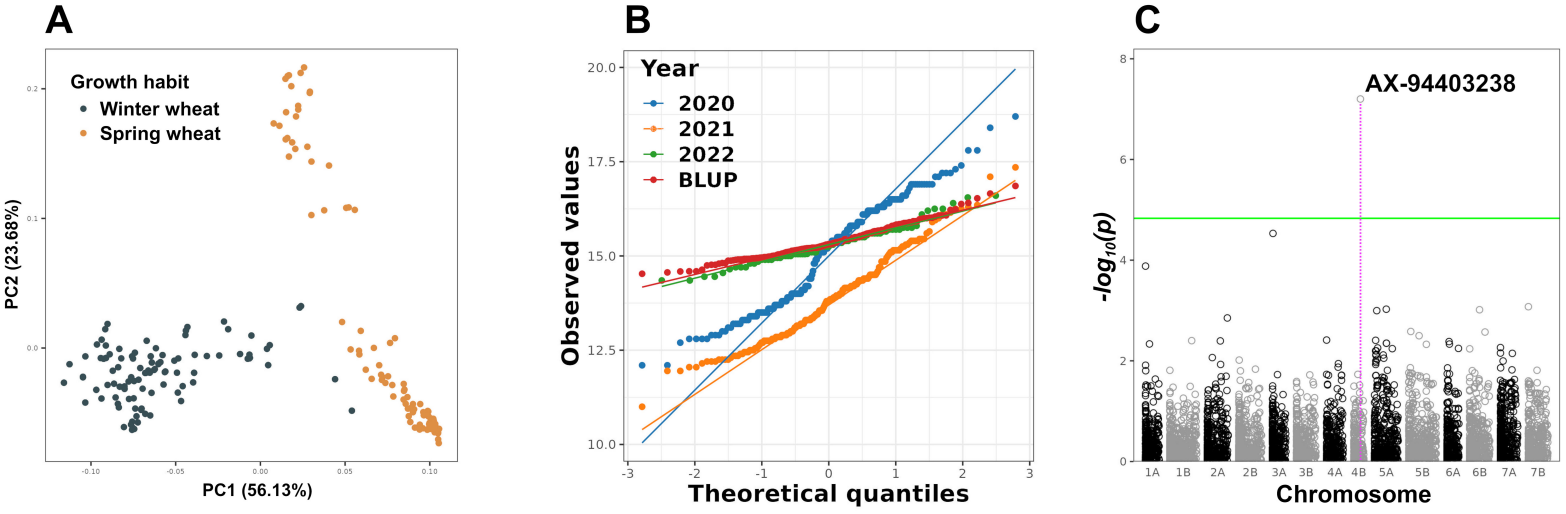
Population structure PCA plot of the durum wheat panel used for GWAS **(A)**. Q-Q plot for the GPC distribution of the durum wheat panel in individual years, as well as the BLUP values (calculated usnig Equation 2), used in GWAS **(B)**. Manhattan plot of GWAS results for GPC. The green line indicates the threshold of statistical significance FDR 5% **(C)**. PCA, Principal Component Analysis; FDR, False Discovery Rate.

A polymorphic panel consisting of 190 accessions of spring and winter durum wheat was used in the GWAS (**Figure 1A**). Distribution of GPC in different growing seasons as well as BLUPs was evaluated using the Q-Q plot and found to be approximately normal (**Figure 1B**). The estimated broad-sense heritability, calculated using Equation 1, of GPC was 0.77, indicating that 77% of the observed phenotypic variation is due to genotype variation.

After genotyping and removing low-quality SNPs, 4927 high-quality SNPs were used in the GWAS. The variance inflation factor was used to correct for observed inflation of P-values (**Supplementary Figure 1**). A single SNP at Chr4B:624,321,301 (AX-94403238, effect size *β* = 0.13, percent of variance explained, PVE = 0.94%) showed a statistically significant association with GPC (**Figure 1C**). The SNP AX-94403238 was annotated as intergenic in the durum wheat reference genome assembly Svevo.v1 (Maccaferri et al., 2019). There were no QTLs or genes previously associated with GPC at these coordinates on chromosome 4B of *T. durum* in the literature. The *serine acetyltransferase 2* gene (*sat2*; TRITD4Bv1G186970) was identified as the closest gene to the SNP AX-94403238 within a genomic region delineated by an average LD decay range of 4.25 Mbp (**Supplementary Figure 2**). According to GO annotation, *sat2* is involved in three biological processes: cysteine biosynthetic process from serine (GO:0006535), acyl-carrier-protein biosynthesis (GO:0031108), and cellular amino acid biosynthesis (GO:0008652). Furthermore, a previous study showed that *sat2* overexpression results in the accumulation of free cysteine in storage proteins of legume seeds (Tabe et al., 2010). Transgenic maize lines overexpressing the Arabidopsis *sat2* gene demonstrated increased levels of storage proteins, characterized by high levels of the sulfur-containing amino acid methionine (Xiang et al., 2018, 2022). Based on proximity to statistically significant SNP, GO annotation and literature evidences, the *sat2* was identified as a candidate gene associated with GPC.

The *sat2* gene sequences from six durum wheat accessions used for GWAS as well as those extracted from genome assemblies of bread wheat, *Tritcum dicoccoides*, and *Aegilops* sp*eltoides* were used for polymorphism mining. A set of KASP markers has been developed for polymorphisms identified in the *sat2* gene sequences (**Supplementary Table 1**).

**Figure 2:**
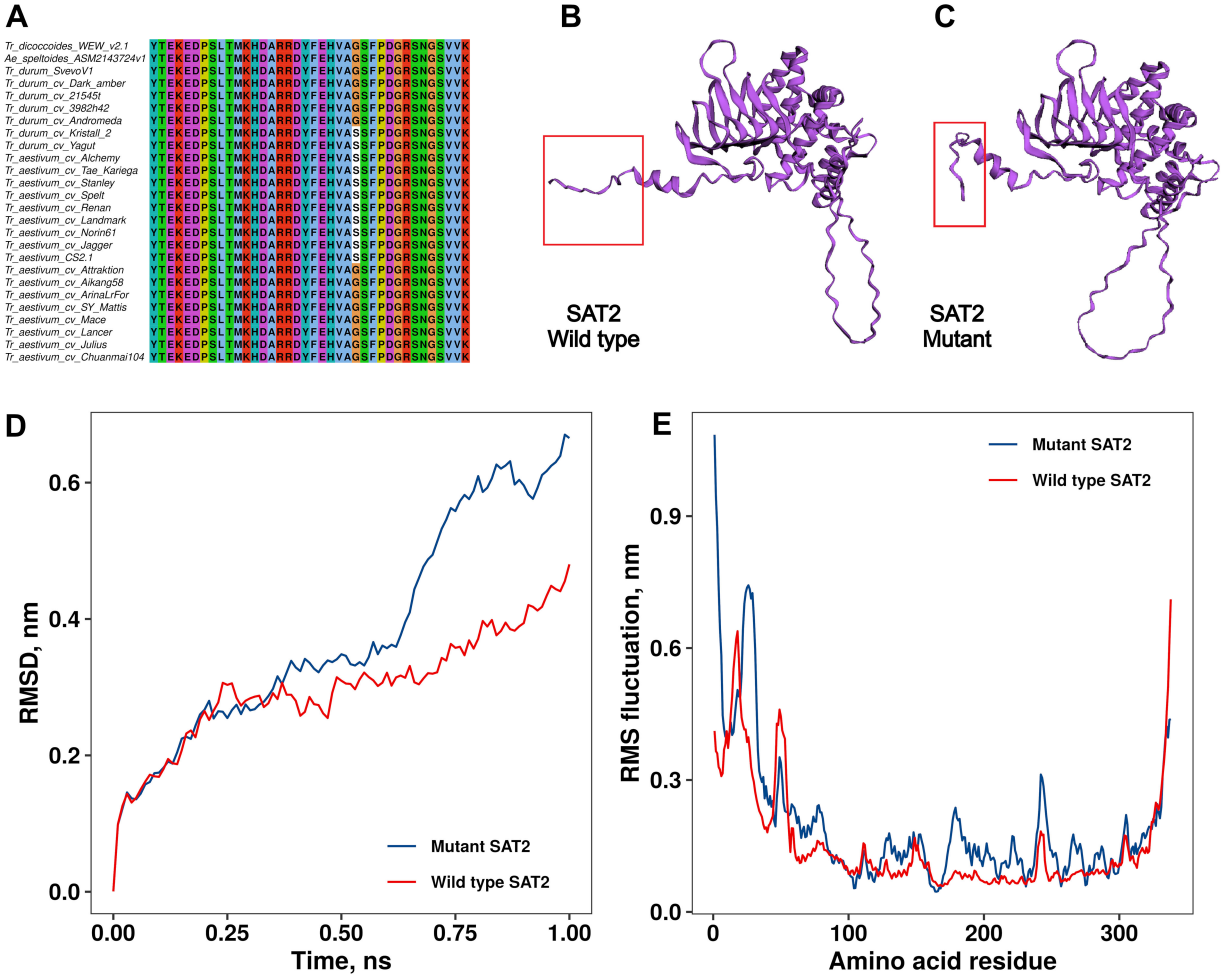
Multiple sequence alignment of the C-terminus of the SAT2 protein from durum wheat, bread wheat, and wild relatives **(A)**. Comparison of the 3D protein structure between wild type **(B)** and mutated **(C)** SAT2 proteins. The difference in the structure due to the single amino acid change is highlighted with red rectangles. Differences in wild type and mutant SAT2 protein conformations stability and dynamics measured by RMSD **(D)** and RMSF **(E)**. RMSD, Root Mean Square Deviation; RMSF, Root Mean Square Fluctuation.

A missense mutation leading to an amino acid change (Gly325Ser) at the C-terminus of the SAT2 protein was identified in the ninth exon of the *sat2* gene (**Figure 2A**). The comparative structural analysis of wild-type (**Figure 2B**) and mutant (**Figure 2C**) SAT2 revealed an extended *α*-helix at the C-terminus in the mutant SAT2.

### 3.2 Mutant SAT2 exhibits reduced global stability and altered local dynamics

RMSD (root mean square deviation) analysis of structural stability revealed greater conformational fluctuations in the SAT2 mutant compared to the wild-type protein. Over the course of 1 ns, the wild-type structure showed RMSD values ranging from 0.1 to 0.45 nm, consistent with moderate global deviations likely attributable to a flexible C-terminal tail. In contrast, the mutant variant reached RMSD values up to ∼0.7 nm, indicating a reduced capacity to maintain its minimized structure (**Figure 2D**). RMSF (root mean square fluctuation) profiles (**Figure 2E**) further highlighted increased mobility in the N-terminal and central regions of the mutant, despite a localized rigidity at the C-terminus that was consistent with previous visual inspections of the trajectory (**Supplementary Data 1**).

These trends suggest that the G325S mutation induces long-range destabilizing effects beyond its immediate vicinity. Additionally, higher minimized potential energy in the mutant (**Supplementary Figure 3**) suggests persistent internal strain despite energy relaxation.

### 3.3 Developed KASP markers for the *sat2* revealed association with GPC

**Figure 3:**
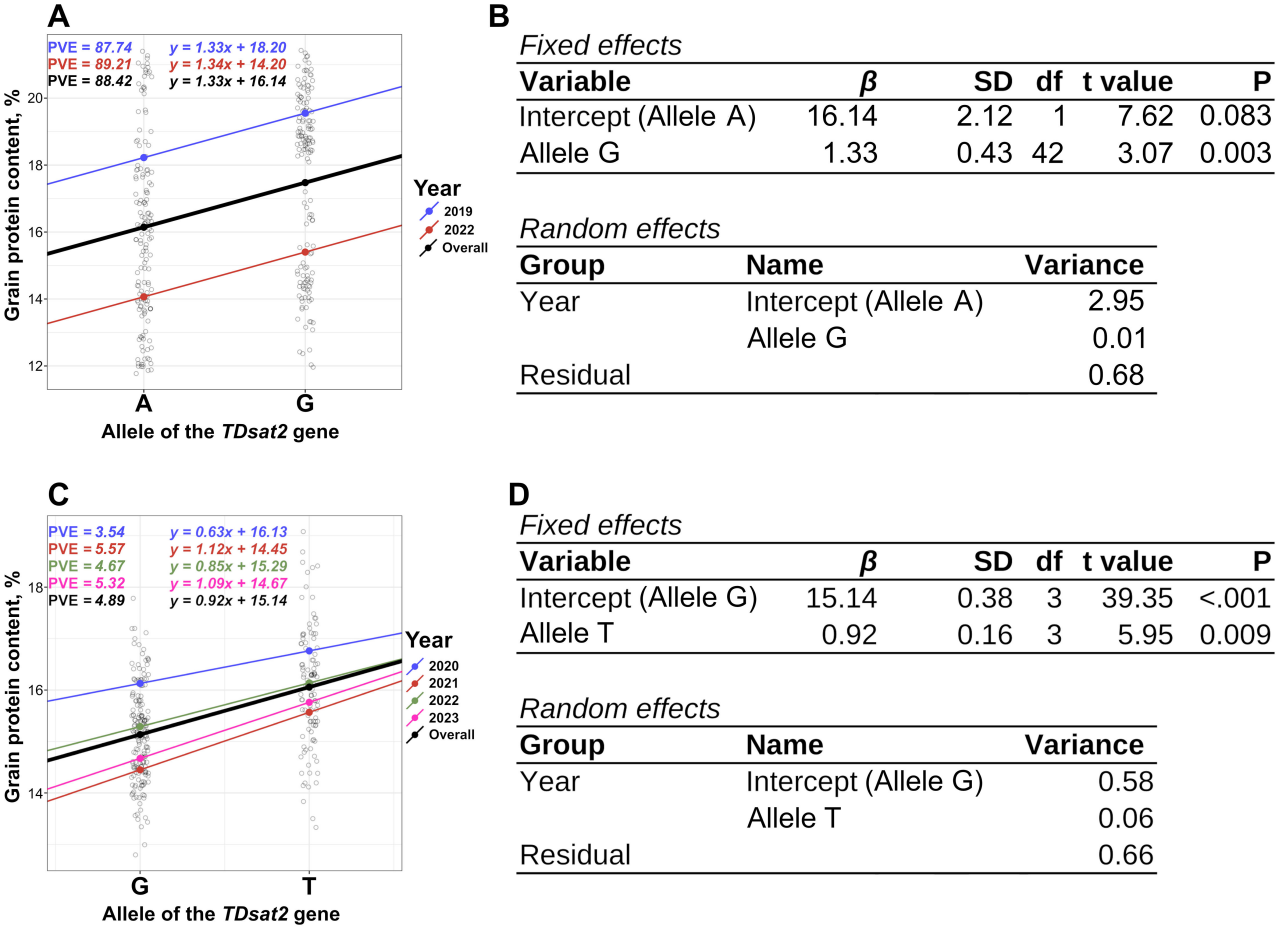
Results of the association analysis between the allelic state of the KASP marker TDsat2.e9.1 and GPC data from the RILs panel of spring durum wheat. PVE was calculated using Equation 3. **(A, B)**. Results of the association analysis between the allelic state of the KASP marker TDsat2.i1.1 and the multi-year GPC data for the panel of spring durum wheat accessions **(C, D)**. RIL, Recombinant Inbred Line; GPC, Grain Protein Content; KASP, Kompetitive Allele Specific PCR.

The KASP marker TDsat2.e9.1 was designed for the identified missense mutation in the ninth exon of the *sat2* (G*>*A), and exhibited good allelic discrimination (**Supplementary Figures 4**). A 1.33% increase in GPC was consistently associated with the G allele of the *sat2* gene in durum wheat RILs (**Figures 3A, B**, **Supplementary Figure 5**; P-value = 0.003).

The KASP marker TDsat2.i1.1 was designed for the SNP (G*>*T) in the first intron of the *sat2* gene, exhibited good allelic discrimination (**Supplementary Figure 6**), however it was found to be monomorphic in RILs; thus, its effect cannot be evaluated using this panel. Validation of the KASP marker TDsat2.i1.1 was performed using an extended panel of durum wheat consisting of 230 winter and spring accessions, including a panel used for GWAS (**Supplementary Figure 7**). A 0.63–1.12% (0.92% in average) increase in GPC depending on the year, was consistently associated with the T allele of the *sat2* gene in spring durum wheat across multiple years (**Figures 3C, D**). ANOVA showed statistically significant differences in GPC between groups of durum wheat accessions carrying G or T alleles of the *sat2* gene (**Figures 3C, D**, **Supplementary Table 2**; P-value *<* 0.001). TDsat2.i1.1 explained a higher proportion (4.89%) of GPC variation than AX-94403238 (0.94%) in spring durum wheat.

ANOVA for winter wheat accessions showed a statistically not significant association between the TDsat2.i1.1 allele and GPC (**Supplementary Figure 8**, **Supplementary Table 2**; P-value = 0.57), as well as an analysis of the complete dataset (**Supplementary Figure 9**, **Supplementary Table 2**; P-value = 0.30). A clear trend was detected showing higher GPC for the T allele of TDsat2.i1.1 (**Supplementary Figure 8**, **Supplementary Table 2**; P-value = 0.30).The A-allele of the KASP marker TDsat2.e9.1 was greatly underrepresented; thus, its effect cannot be assessed using this panel.

### 3.4 *sat2* is predominantly expressed in grains

Comparative transcriptomics approaches are crucial in transferring biological information, particularly conserved gene functions from better-studied species (Zuluaga et al., 2023). This approach was previously used for the identification of candidate genes based on both functional annotation and expression profiles in the closely related species *Triticum aestivum* (Desiderio et al., 2019). Although omics resources for durum wheat are steadily increasing, more extensive data, resources, and databases are available for bread wheat due to its greater economic importance (Zuluaga et al., 2023).

**Figure 4:**
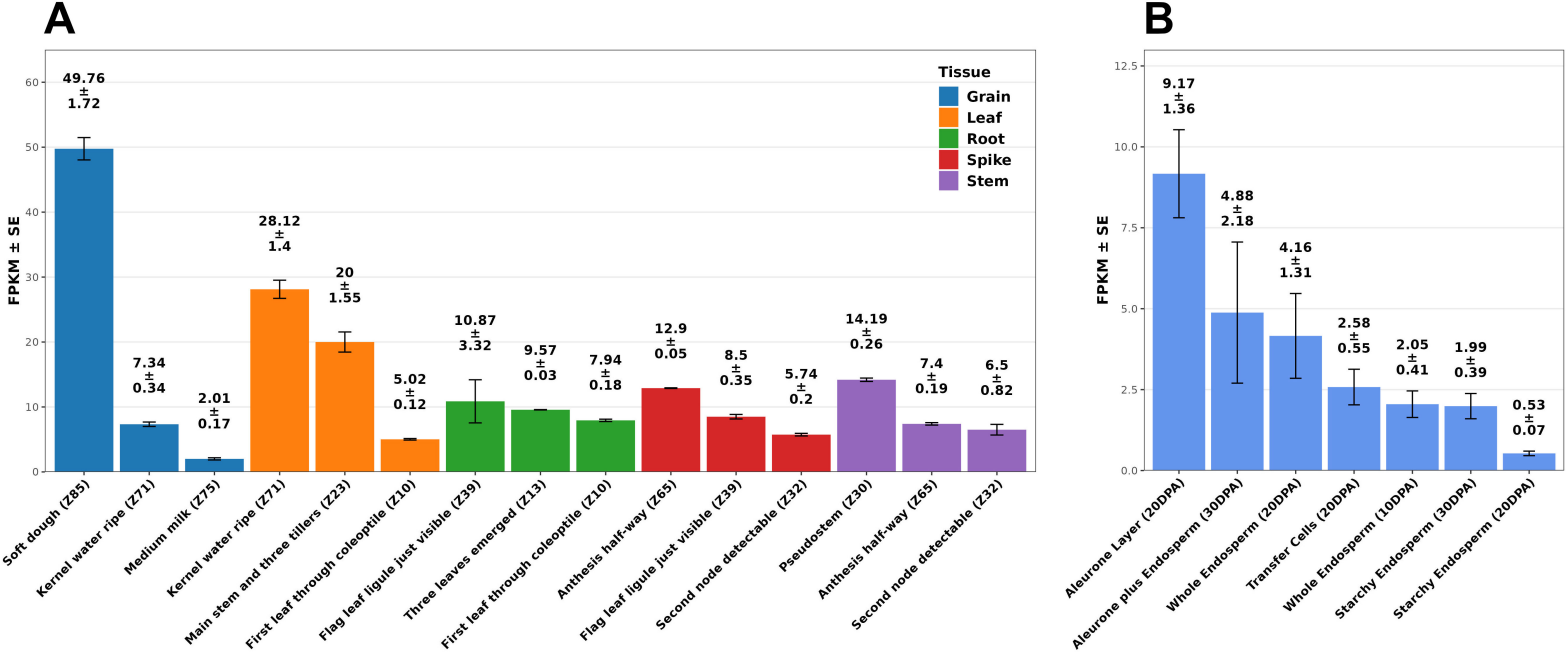
The expression profile of the durum wheat *sat2* gene homologous in bread wheat (*TAsat2*) in different tissues at different developmental stages **(A)**. The expression profile of the durum wheat *sat2* gene homologous in bread wheat in different tissues of grain at different developmental stages **(B)**. Expression levels are presented as FPKM. Error bars are expressed as SE. Data were extracted from the WheatExp database (Pearce et al., 2015). Developmental stages follow the Zadoks growth scale (Zadoks et al., 1974). DPA, days post-anthesis; SE, standard error; FPKM, Fragments Per Kilobase of transcript per Million mapped reads.

The expression profile of the *sat2* homologous from bread wheat (*TAsat2*) revealed that its transcription levels were highest in grains during the “soft dough” stage and in leaves during at the “kernel water ripe” and “main stem and three tillers” stages (**Figure 4A**). *TAsat2* expression in grains was most pronounced in the aleurone layer and sub-aleurone cells at 20–30 days post-anthesis (**Figure 4B**). Therefore, mutations in SAT2 may affect the availability of cysteine in aleurone and subaleurone cells, change protein synthesis, and shift the protein gradient in the starchy endosperm, contributing to the observed variation in GPC.

## 4 Discussion

In this study, we identified the *sat2* gene and demonstrated that G-allele of TDsat2.e9.1 and T-allele of the TDsat2.i1.1 are associated with GPC in spring, but not winter, durum wheat. Members of the SAT gene family encode serine acetyltransferase (SAT/SERAT), a key enzyme in cysteine synthesis. SAT is a critical component of the cysteine synthase complex, a hetero-oligomer comprising SAT and O-acetylserine-(thiol)lyase (OASTL) proteins (Wirtz and Hell, 2006). In plant tissues, OASTL is present in large excess relative to SAT, rendering SAT concentration a limiting factor for complex formation (Haas et al., 2008; Wirtz and Hell, 2006).

The KASP marker TD2.i1.1 targets a mutation in the first intron of *sat2* without altering the encoded protein’s amino acid sequence. Previous studies indicate that most variants in the first intronic region influence gene expression (Kowal et al., 2024). Thus, the intronic SNP identified in *sat2* could elevate its expression, which, in turn, might increase cysteine abundance in grain storage proteins (Tabe et al., 2010), suggesting a potential role in transcriptional regulation.

The KASP marker TDsat2.e9.1 targets a missense mutation (Gly325Ser) in the ninth exon of *sat2*, which induces structural changes in the SAT2 C-terminus. The C-terminal domain of a SAT mediates protein-protein interactions with OASTL, forming the structural basis of the hetero-oligomeric cysteine synthase complex. Substitutions in this region reduce or abolish SAT enzymatic activity (Wirtz et al., 2001). Molecular dynamic analysis in the wild-type SAT2 revealed increased flexibility of the C-terminal tail facilitates moderate global deviations (0.1–0.45 nm RMSD). By contrast, the SAT2 mutant exhibits substantially larger global displacements (up to ∼0.7 nm RMSD) and a rigidified C-terminus, accompanied by heightened mobility in the N-terminal and central regions. These results indicate that the G325S mutation leads to an overall less resilient conformation.

Based on spatiotemporal patterns of *sat2* expression, the grain filling stage exhibits the highest expression levels and coincides with the beginning of protein accumulation in grains (Zhang et al., 2021). Furthermore, during the grain filling, the amino acids produced by proteolysis in the leaves are transported to the grain as substrates for protein synthesis (Fisher and Gifford, 1986), which explains the elevated *sat2* transcript levels in leaves. The observed differences in the contribution of the same *sat2* genotype to GPC in spring and winter durum wheat could result from unique biotic and abiotic factors during grain formation and maturation in different seasons of the year.

The KASP markers TDsat2.i1.1 and TDsat2.e9.1 represent practical tools for spring durum wheat breeding programs. Although no statistical difference was observed in winter durum wheat, the *sat2* marker could be used in breeding programs focusing on pyramiding genes involved in GPC or other agronomically important traits, such as plant height and frost resistance. In winter durum wheat breeding, concurrent selection for frost tolerance and grain quality is critical, as genetic analyses confirm these traits can be combined without trade-offs (Longin et al., 2013). Furthermore, the semi-dwarfism gene *Rht* exhibits a pleiotropic effect, leading to reduced grain protein content in durum wheat (Achilli et al., 2022).

The identified mutations in the *sat2* may regulate GPC through distinct mechanisms: the missense exonic mutation alters SAT enzymatic activity, while the intronic mutation potentially affects transcriptional regulation. Field data robustly associated *sat2* allelic variation with differential protein accumulation, providing a foundation for breeders to leverage this gene in GPC improvement. While this study does not elucidate the precise mechanisms by which *sat2* influences GPC in durum wheat, the evidence presented suggests that the identified SNPs are likely causal variants.

## Data Availability

The RNA-seq datasets analyzed for this study are publicly available via the WheatExp database. Details are provided in the **Supplementary Methods**.
