# Supplementary material for: High-quality bonds: serine acetyltransferase 2 gene revealed by GWAS is associated with grain protein content in spring durum wheat
Source: Front Plant Sci. 2025 Aug 12;16:1632673. doi: 10.3389/fpls.2025.1632673 (PMC12378379; doi:10.3389/fpls.2025.1632673)
Supplement: Supplementary file 1 [file DataSheet1.pdf]

Supplementary Material

1 SUPPLEMENTARY DATA

1.1 Estimation of linkage disequilibrium decay

Linkage disequilibrium (LD) between all pairs of markers was assessed using plink2 v2.00a5LM (Chang et al., 2015). LD was calculated as the square of allelic correlation frequency (r^2). Background LD was calculated as the 95th percentile value of the LD distribution of unlinked SNPs on different chromosomes. Pairwise distances between markers on the same chromosome were plotted against LD, and the regression curve was calculated by the LOESS (locally estimated scatterplot smoothing) method (Hastie, 2017). The critical distance between markers at which LD decays was calculated as the point of intersection of the LOESS regression curve and the straight line corresponding to the background LD. Pairs of SNPs that have an r^2 value above this threshold are considered physically linked. The critical distance was calculated for the entire genome as well as separately for the A- and B-sub genomes.

1.2 Secondary and Tertiary Protein Structure Prediction

The structural prediction of the SAT2 protein was conducted using computational tools. For tertiary structure prediction, AlphaFold3 generated a 3D structural model (`https://alphafoldserver.com/`, accessed on March 4, 2025), which was visualized and analyzed using UCSF Chimera 1.18 (`https://www.cgl.ucsf.edu/chimera/`, accessed on March 4, 2025).

1.3 Computational prediction of a protein mutation effect

The effect of the G325S mutation was evaluated through molecular dynamics (MD) simulations, conducted in accordance with established protocols outlined in (Collier et al., 2020) and (Huang et al., 2018).

The initial 3D structures of the wild-type (G325) and mutant (S325) SAT2 proteins were processed using GROMACS 2024.5 (`www.gromacs.org`, accessed on June 25, 2025). Each structure was first converted into a force field-compliant format with the OPLS-AA/L (Optimized Potentials for Liquid Simulations – All Atom, with torsion parameters refitted using LMP2) and the SPC/E (Simple Point Charge-Extended) water model. The proteins were placed in a cubic box with a minimum distance of 1 nm from the box edges, followed by solvation with the spc216 (Simple Point Charge) water model. To neutralize the net positive charge, counter-ions (Cl^-) were added in the solvated system.

Production MD simulations were performed for 1 ns at 300 K and 1 bar, with system coordinates recorded every 1 picoseconds (ps). Although brief, trajectories of this duration have been shown to achieve initial conformational relaxation and capture relevant dynamic behavior in all-atom MD studies (Karami et al., 2018). To eliminate steric clashes and unfavorable contacts prior to dynamics, simulated protein systems were first energy-minimized using the steepest descent algorithm, followed by a two-phase equilibration lasting 100 ps each to ensure stable temperature and pressure conditions (Supplementary Figure 10A-C). The first phase employed a constant-volume (NVT) ensemble using the velocity-rescaling thermostat. The second phase was conducted under a constant-pressure (NPT) ensemble using the Parrinello-Rahman barostat. Structural stability was assessed via root mean square deviation (RMSD) of the backbone atoms across the entire 1-ns trajectory. Additionally, residue-level fluctuations were evaluated via root mean square fluctuation (RMSF).

The visualization of protein dynamics was performed using the DynaMut2 web server (`www.biosig.lab.uq.edu.au/dynamut2/nma`, accessed on June 25, 2025) with the REACH approach, which integrates normal mode analysis (NMA) to assess the impact of point mutations on structural flexibility.

1.4 Genotyping of winter and spring durum wheat population using developed KASP markers

Genotyping of durum wheat was conducted using developed KASP markers in a 384-well PCR plate. A 5 μL PCR mixture comprised 2.5 μL of KBS-1050-102 Master Mix, which contains FAM, HEX, and ROX dyes (LCG Biosearch Technologies, UK); 0.75 pM of each allele-specific primer and 1.875 pM of the common primer; and 50 ng of template DNA. The subsequent touchdown PCR protocol was employed: 15 min at 94 °C (hot start); 20 s at 94 °C followed by 60 s at 61–55 °C (temperature was decreased by 0.6 °C in each successive cycle); 30 s at 94 °C (for 10 cycles); 20 s at 94 °C followed by 60 s at 55 °C (for 35 cycles); signal reading conducted for 60 s at 37 °C. PCR was conducted using the BIO-RAD CFX96 (Bio-Rad Laboratories, Inc., Hercules, CA, USA). Subsequently, results were analyzed using Bio-Rad CFX Manager 3.1 software.

1.5 Evaluation of spatial and temporal expression patterns of *sat2* gene

The RNA sequencing datasets were used to investigate the transcription of identified candidate genes in different wheat grain tissues (Pearce et al., 2015a) and phases of plant development (Choulet et al., 2014). Differential expression data for candidate genes was extracted from ”WheatExp: An Expression Database for Polyploid Wheat” (Pearce et al., 2015b) and visualized using the ggplot2 package in R v4.3.1. The ”WheatExp” database contains only hexaploid bread wheat expression data. Corresponding transcripts of the candidate genes from bread wheat were identified using BLASTn integrated into the ”WheatExp” database with an E value threshold of 1e-10. The highest-scoring transcripts located on the same chromosome as the candidate genes were selected for expression analysis. Developmental stages follow the Zadoks growth scale (Zadoks et al., 1974).

2 SUPPLEMENTARY TABLES AND FIGURES

2.1 Figures

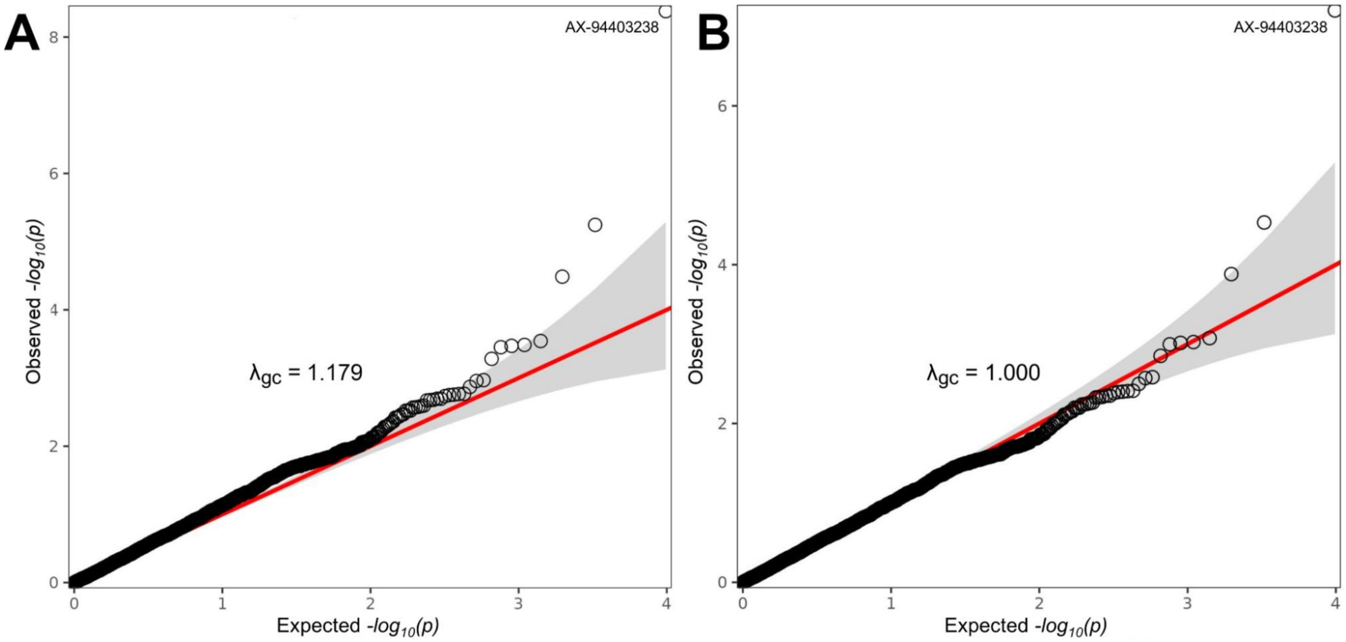

Figure S1. The Quantile-Quantile (Q-Q) plot illustrates the correspondence between the distribution of observed P-values and the expected distribution. P-values are represented as $-log10(P)$. **(A)** Q-Q plot prior to inflation adjustment of P-values (λ_{gc} = 1.179); **(B)** Q-Q plot following inflation correction of P-values using VIF (λ_{gc} = 1.000).

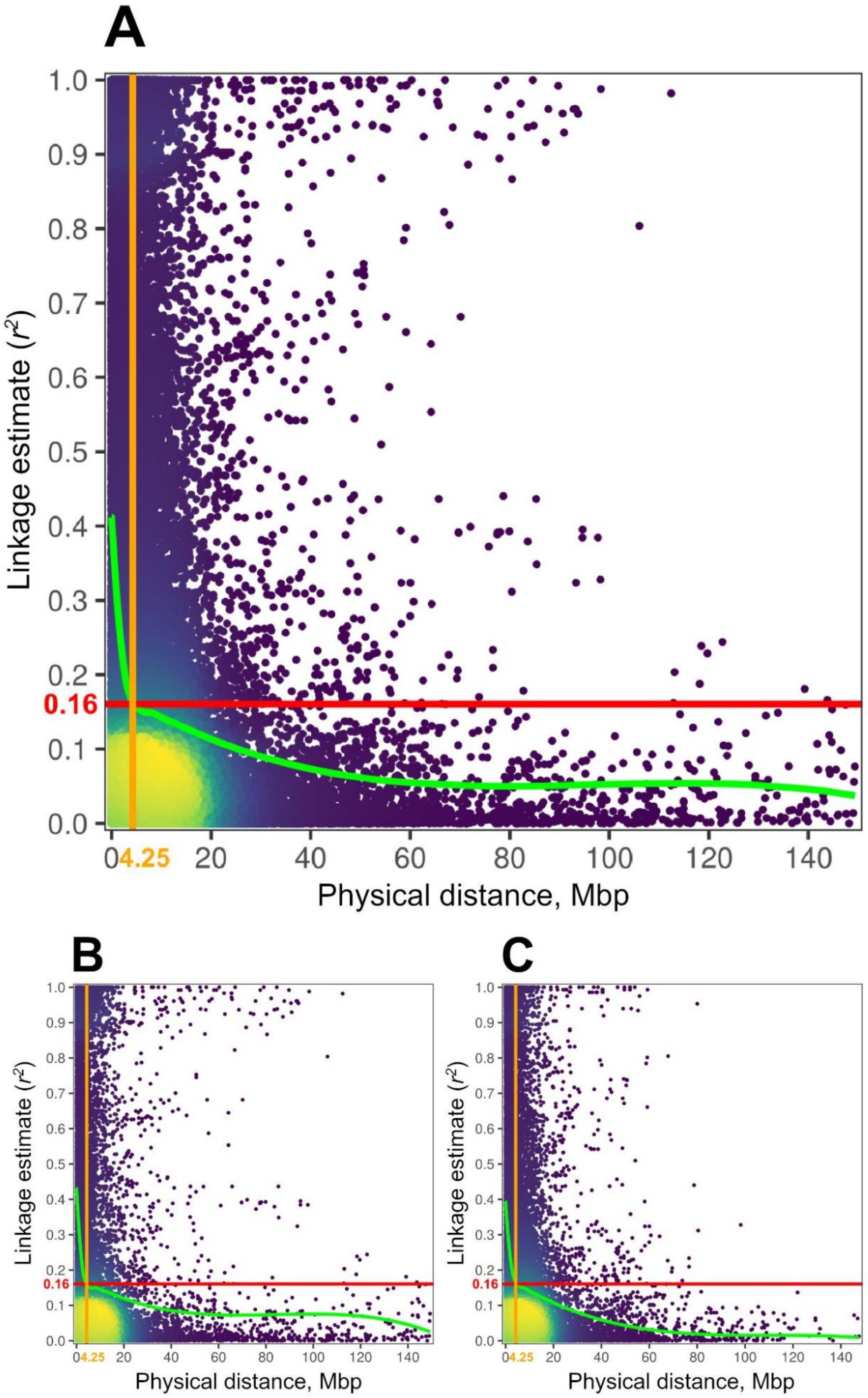

Figure S2. A genome-wide LD decay plot **(A)**, as well as on the A sub-genome **(B)** and B sub-genome **(C)** separately. The physical distance between markers, measured in millions of base pairs (Mbp), is plotted against the LD (r^2) estimated for each SNP pair on the same chromosome. The red line represents the background LD, calculated as the 95th percentile of the r^2 distribution for unlinked SNPs across different chromosomes. SNP pairs that fall above the red line are considered physically linked. The green line denotes the regression curve derived using the LOESS method. The orange line indicates the critical physical distance between SNPs at which the regression curve intersects the background LD threshold.

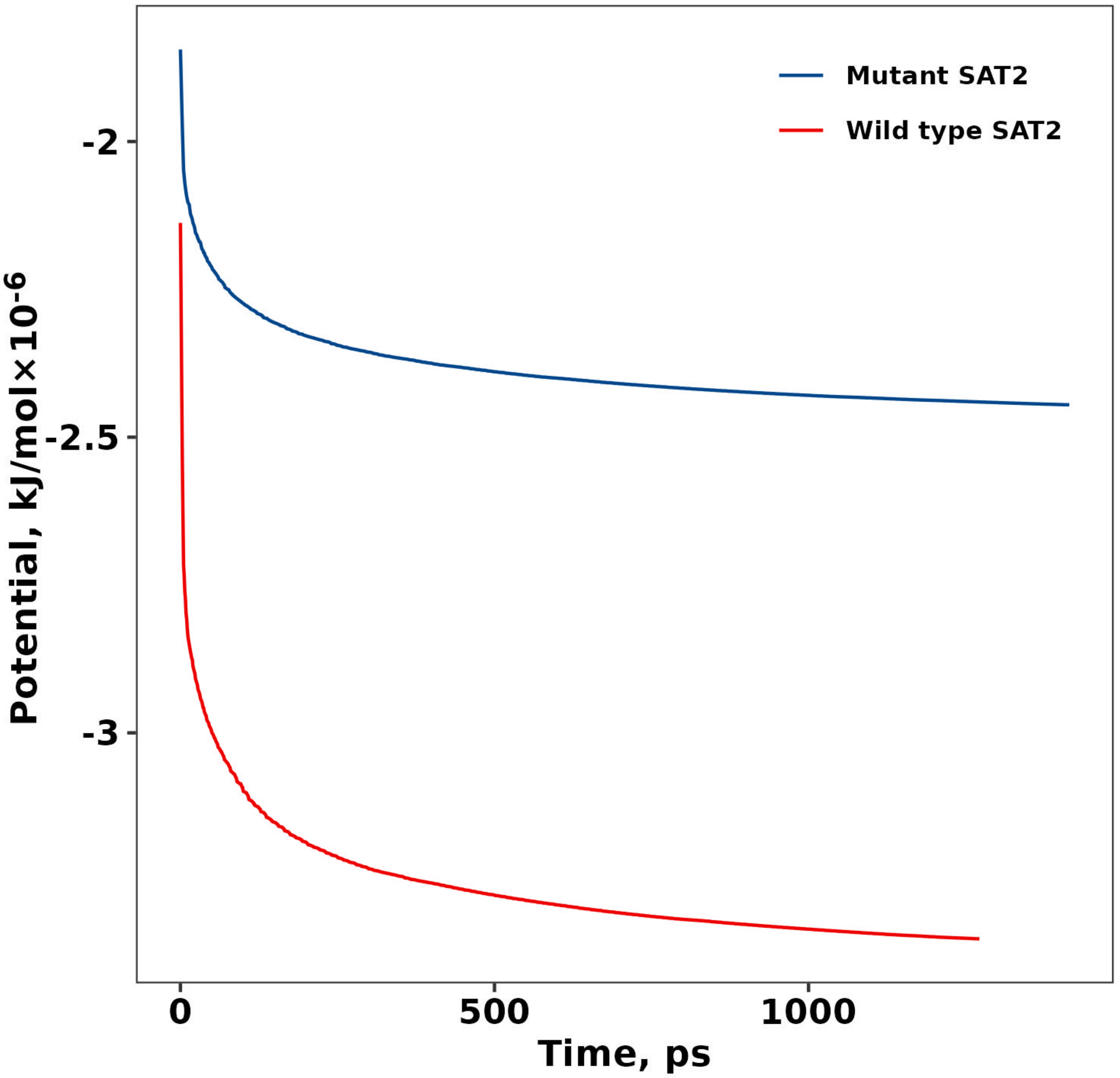

Figure S3. Time evolution of potential energy (kJ/mol$\times 10^{-6}$) for wild type and mutant SAT2 proteins up to 1 ns.

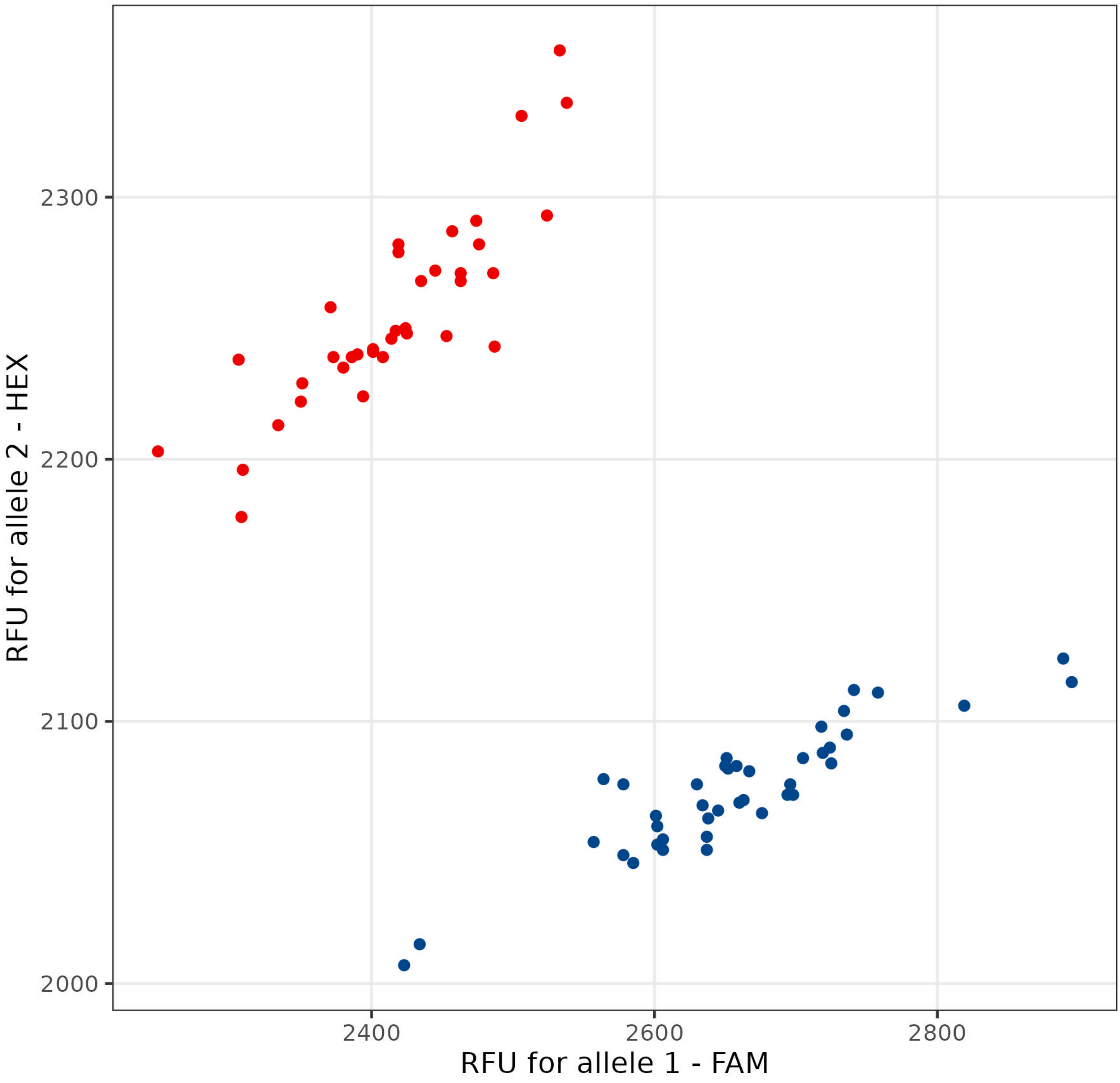

Figure S4. The genotyping results of the durum wheat RILs panel obtained using the developed KASP marker TDsat2.e9.1. Red dots indicate accessions with one allele of the *sat2* gene in homozygous form (HEX), while blue dots indicate an alternative allele of the *sat2* gene in homozygous form (FAM).

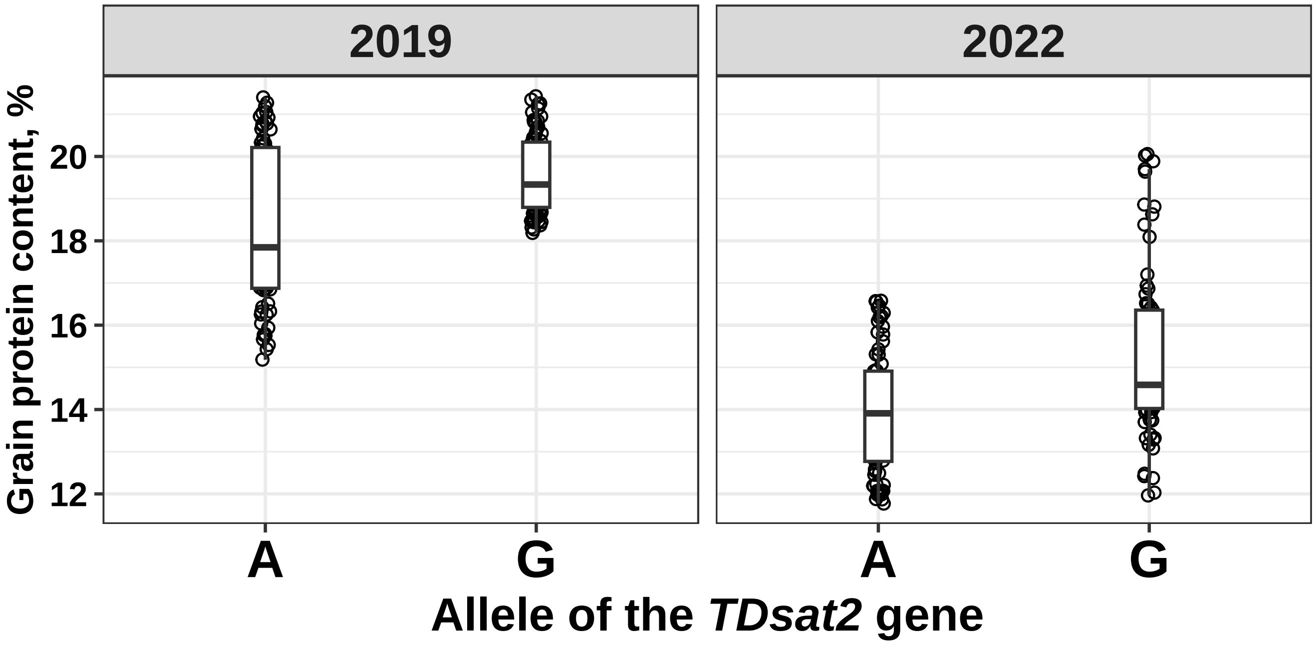

Figure S5. Box plots with individual data points showing GPC across growing years for durum wheat accessions from a RIL population, used to validate the TDsat2.e9.1 KASP marker.

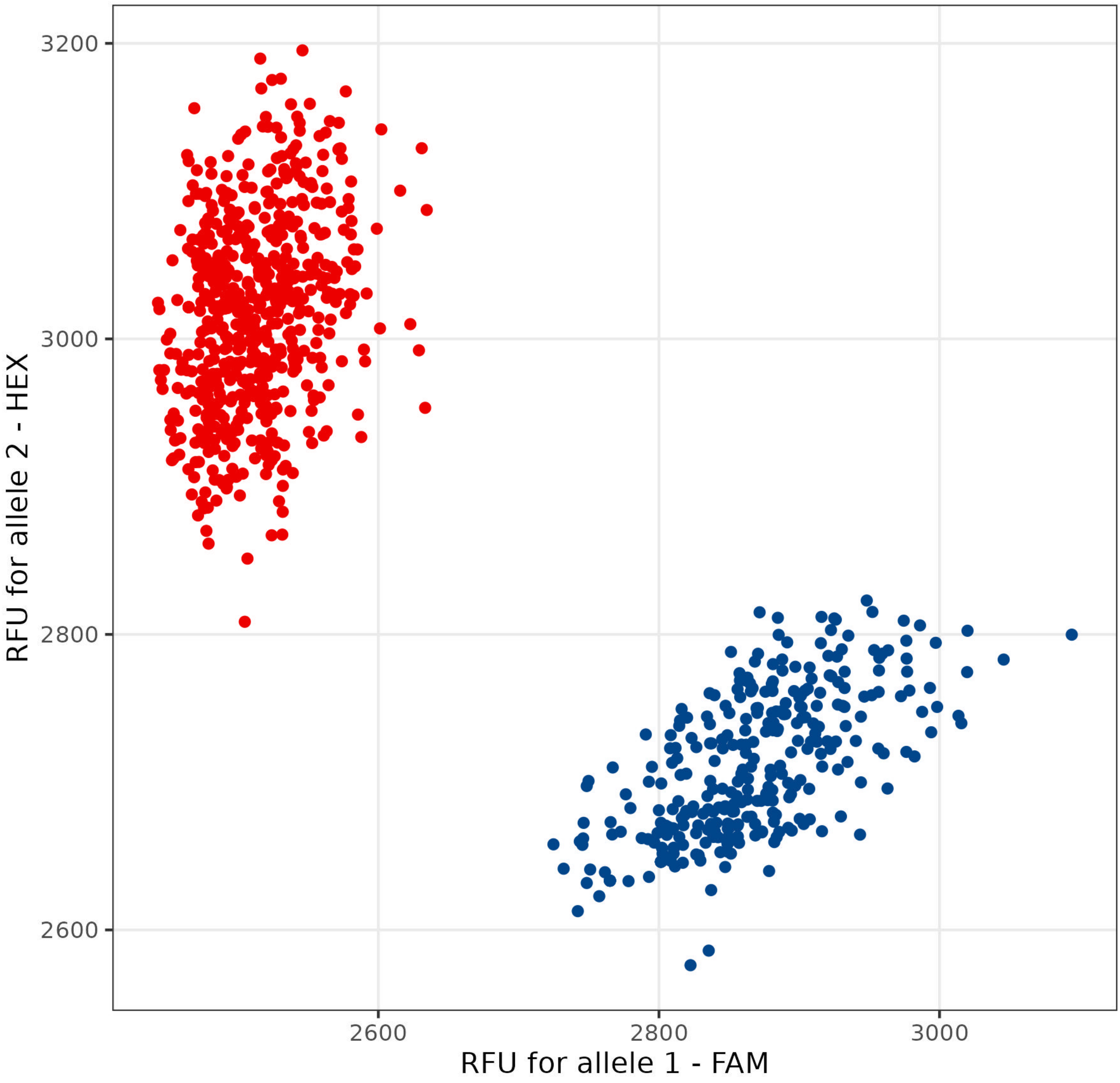

Figure S6. The genotyping results of the durum wheat panel obtained using the developed KASP marker TDsat2.i1.1. Red dots indicate accessions with one allele of the *sat2* gene in homozygous form (HEX), while blue dots indicate an alternative allele of the *sat2* gene in homozygous form (FAM).

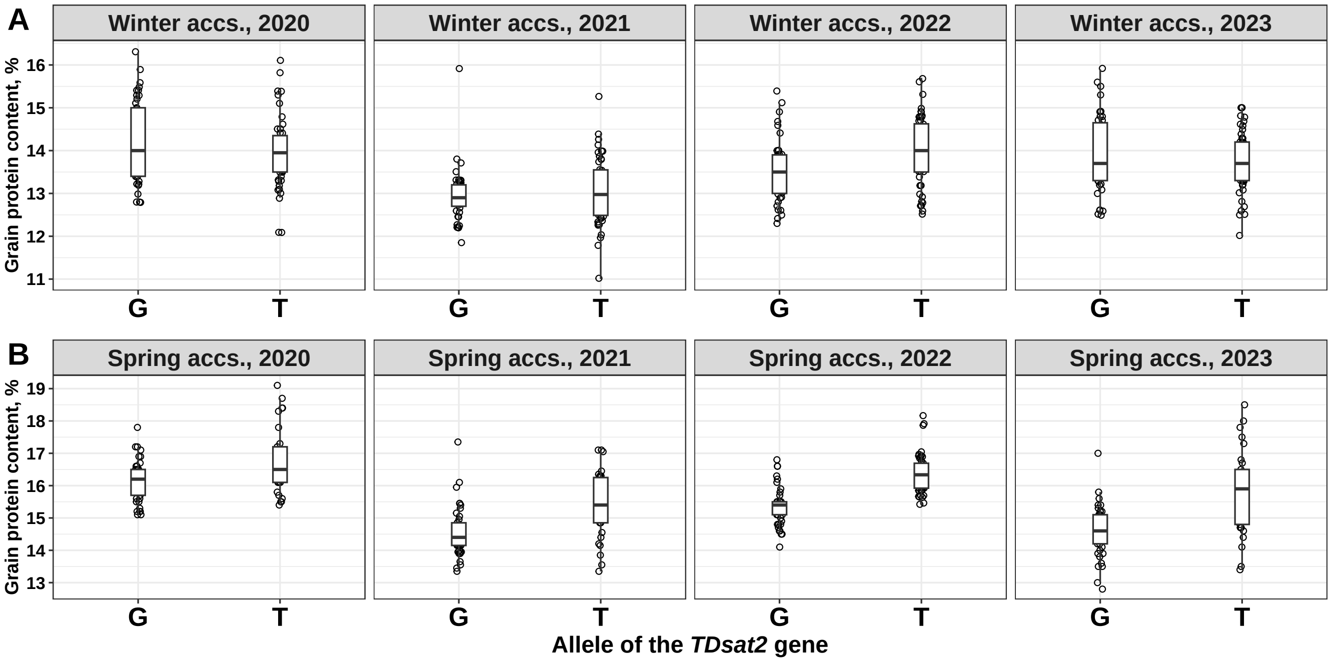

Figure S7. Box plots with individual data points showing GPC across different growing years for winter **(A)** and spring **(B)** durum wheat accessions in the extended GWAS panel used to validate the TDsat2.i1.1 KASP marker.

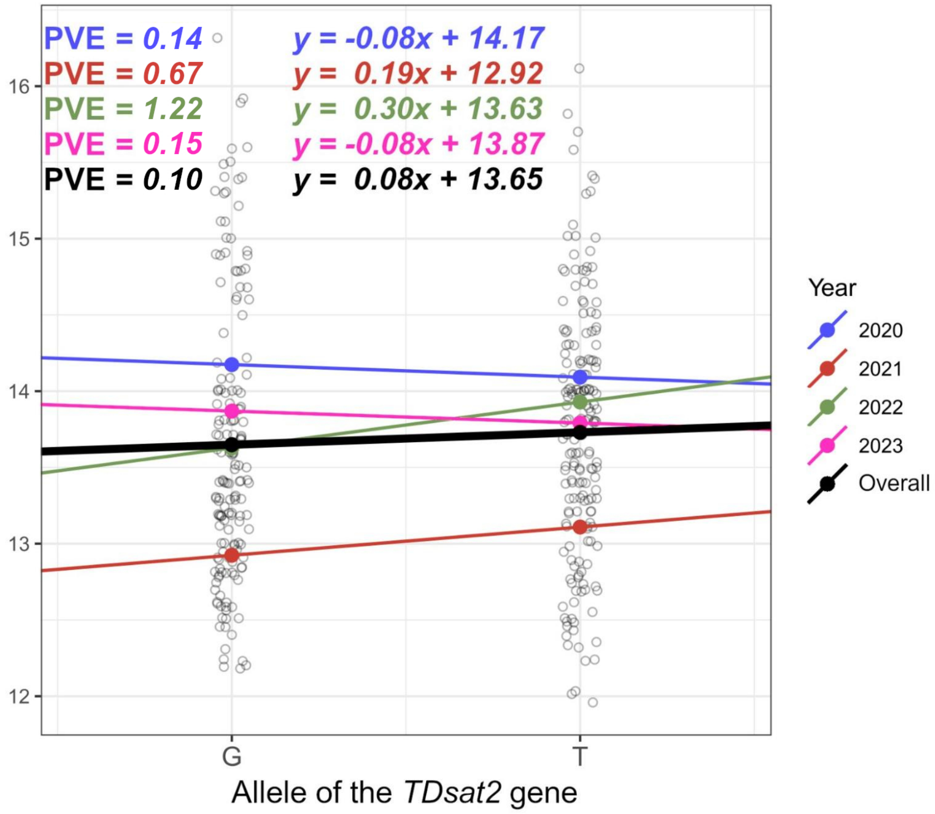

Fixed effects

Variable	β	SD	df	t value	*P*
Intercept	13.65	0.28	3	49.06	<.001
TDsat2(T)	0.08	0.14	3	0.57	0.61

Random effects

Group	Name	Variance
Year	Intercept	0.30
	TDsat2(T)	0.05
Residual		0.57

Figure S8. The results of the analysis of the association between the allelic state of the TDsat2.i1.1 KASP marker and multi-year GPC data for the winter durum wheat varieties, using MLM and the parameters of the constructed models. The data in the tables are based on the full (Overall) model. The coefficients β and PVE are presented in the tables and on plots in phenotypic units (%).

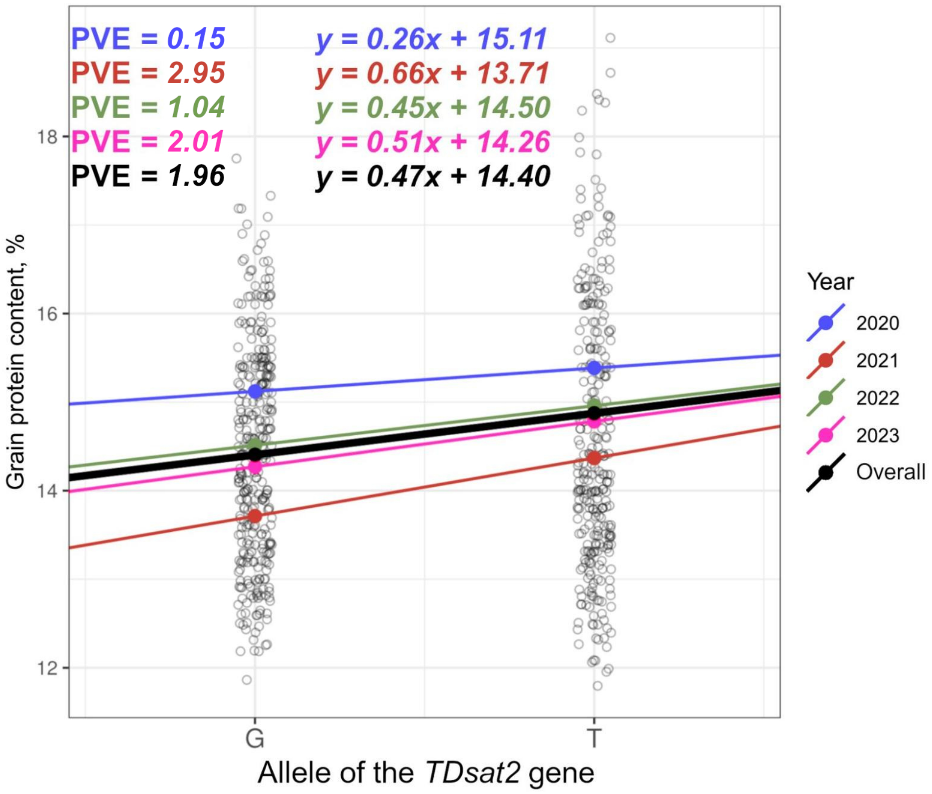

Fixed effects

Variable	β	SD	df	t value	P
Intercept	14.40	0.80	1.32	17.92	0.02
TDsat2(T)	0.47	0.45	1.07	1.05	0.48

Random effects

Group	Name	Variance
Year	Intercept	0.35
	TDsat2(T)	0.03
Type	Intercept	1.12
	TDsat2(T)	0.38
Residual		0.69

Figure S9. The results of the analysis of the association between the allelic state of the TDsat2.i1.1 KASP marker and multi-year GPC data for the complete dataset of winter and spring durum wheat varieties using MLM and the parameters of the constructed models. The data in the tables is based on the full (Overall) model. The data in the tables are based on the full (Overall) model. The coefficients β and PVE are presented in the tables and on plots in phenotypic units (%).

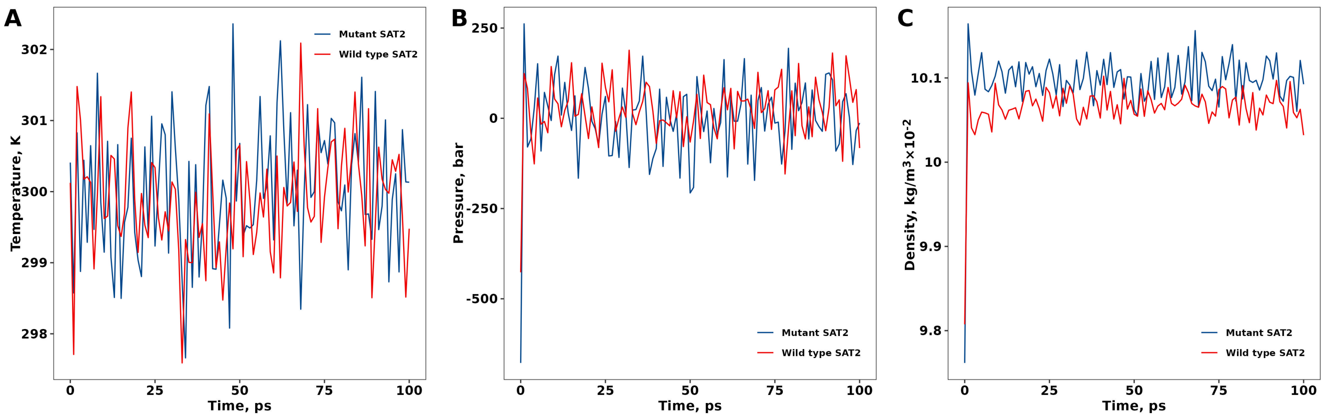

Figure S10. Time dependence of thermodynamic properties during equilibration of wild-type (red) and mutant (blue) SAT2 proteins in MD simulations. Instantaneous average temperatures of wild-type and mutant proteins plotted over 100 ps **(A)**. Instantaneous pressure profiles for wild-type and mutant proteins systems over 100 ps **(B)**. Instantaneous density values as a function of time (ps) for wild-type and mutant SAT2 proteins **(C)**.

2.2 Tables

Table S1. Primers of the KASP markers designed for identified polymorphisms in the TDsat2 gene. Discriminating nucleotides at 3‘-end denoted in the brackets.

Marker name	Discriminating primer (5'–3')	Common primer (5'–3')
TDsat2.e9.1	TAGATTATTTCGAGCATGTTGCC[G/A]	AAACTCACCGTTGGATCTACC
TDsat2.i1.1	GTTTCAGACGGCAAAGAATCCA[T/G]	GTGACTTGAAAGAAGAGAATCCAGC

Table S2. The results of the ANOVA for the association of the allelic state of TDsat2.i1.1 and GPC.

Dataset	Wald χ^2	df	P value
Complete (winter + spring)	1.09	1	0.30
Spring varieties	35.37	1	< 0.001***
Winter varieties	0.33	1	0.57

REFERENCES

Chang, C. C., Chow, C. C., Tellier, L. C., Vattikuti, S., Purcell, S. M., and Lee, J. J. (2015). Second-generation plink: rising to the challenge of larger and richer datasets. *Gigascience* 4, s13742–015

Choulet, F., Alberti, A., Theil, S., Glover, N., Barbe, V., Daron, J., et al. (2014). Structural and functional partitioning of bread wheat chromosome 3b. *Science* 345, 1249721

Collier, T. A., Piggot, T. J., and Allison, J. R. (2020). Molecular dynamics simulation of proteins. *Protein Nanotechnology: Protocols, Instrumentation, and Applications* , 311–327

Hastie, T. J. (2017). *Statistical models in S* (Routledge)

[Dataset] Huang, J., Lemkul, J. A., Eastman, P. K., and MacKerell Jr, A. D. (2018). Molecular dynamics simulations using the drude polarizable force field on gpus with openmm: Implementation, validation, and benchmarks

Karami, Y., Bitard-Feildel, T., Laine, E., and Carbone, A. (2018). “infostery” analysis of short molecular dynamics simulations identifies highly sensitive residues and predicts deleterious mutations. *Scientific reports* 8, 16126

Pearce, S., Huttly, A. K., Prosser, I. M., Li, Y.-d., Vaughan, S. P., Gallova, B., et al. (2015a). Heterologous expression and transcript analysis of gibberellin biosynthetic genes of grasses reveals novel functionality in the ga3ox family. *BMC Plant Biology* 15, 1–19

Pearce, S., Vazquez-Gross, H., Herin, S. Y., Hane, D., Wang, Y., Gu, Y. Q., et al. (2015b). Wheatexp: an rna-seq expression database for polyploid wheat. *BMC plant biology* 15, 1–8

Zadoks, J. C., Chang, T. T., and Konzak, C. F. (1974). A decimal code for the growth stages of cereals. *Weed research* 14, 415–421
