# Supplementary material for: High-quality bonds: serine acetyltransferase 2 gene revealed by GWAS is associated with grain protein content in spring durum wheat
Source: Front Plant Sci. 2025 Aug 12;16:1632673. doi: 10.3389/fpls.2025.1632673 (PMC12378379; doi:10.3389/fpls.2025.1632673)
Supplement: Supplementary file 2 [file Presentation1.pptx]

## Slide 1
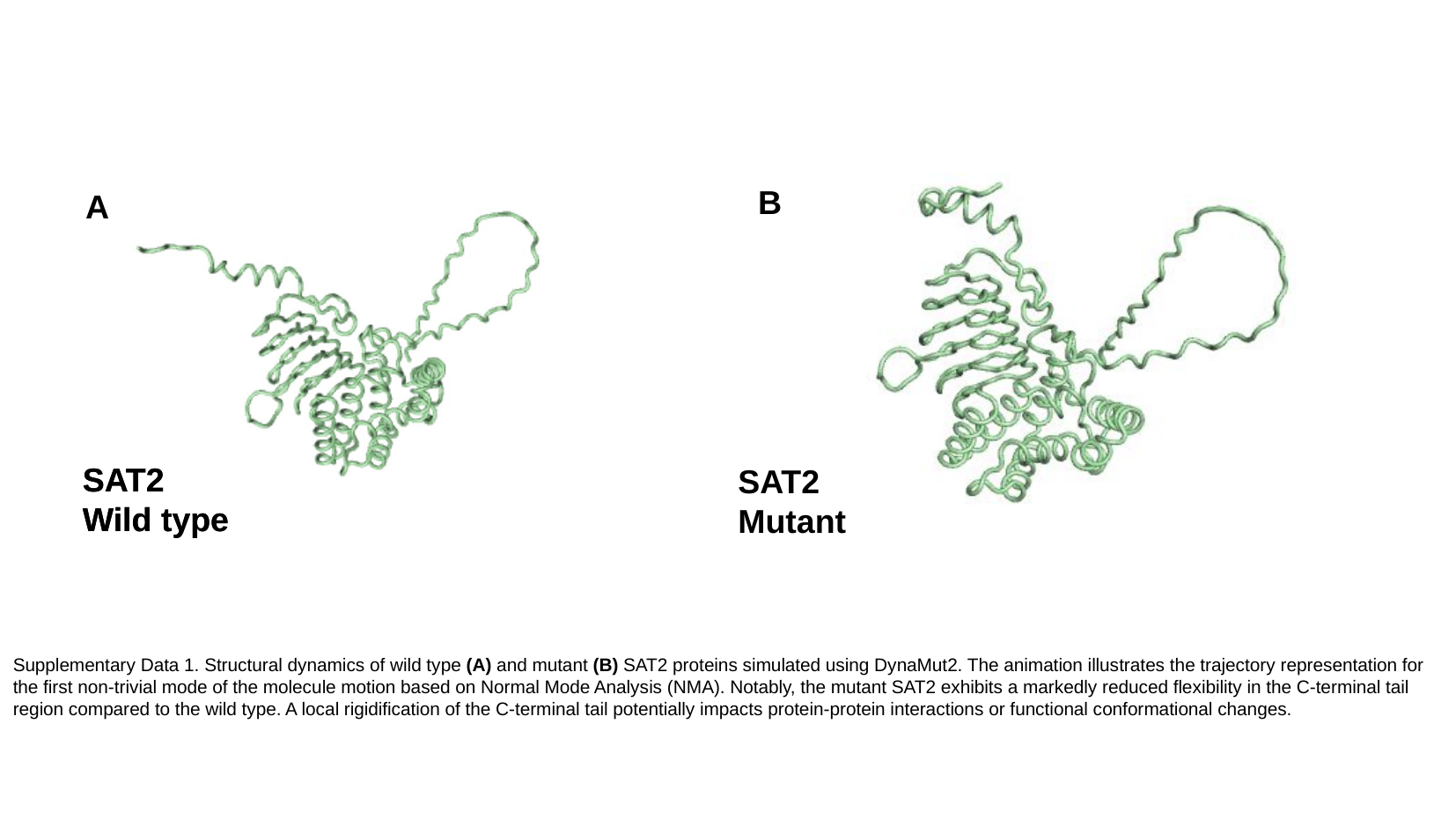

B
A
SAT2
Wild type
SAT2
Wild type
SAT2
Mutant
Supplementary Data 1. Structural dynamics of wild type (A) and mutant (B) SAT2 proteins simulated using DynaMut2. The animation illustrates the trajectory representation for the first non-trivial mode of the molecule motion based on Normal Mode Analysis (NMA). Notably, the mutant SAT2 exhibits a markedly reduced flexibility in the C-terminal tail region compared to the wild type. A local rigidification of the C-terminal tail potentially impacts protein-protein interactions or functional conformational changes.
